# Correlation Between the Body Mass Index and the Distance From the Skin to the Lumbar Epidural Space in Term Parturients

**DOI:** 10.7759/cureus.95124

**Published:** 2025-10-22

**Authors:** Dinesh Tamilselvan, Sarita Ramchandani, Vinita Singh, Monica Khetrapal, Pradeep Khobragade, Chinmaya K Panda

**Affiliations:** 1 Anesthesiology, All India Institute of Medical Sciences, Raipur, Raipur, IND; 2 Obstetrics and Gynecology, All India Institute of Medical Sciences, Raipur, Raipur, IND

**Keywords:** body mass index, labor analgesia, skin to lumbar epidural space, term parturient, ultrasound

## Abstract

Introduction

Placement of lumbar epidural catheters is the gold standard practice for providing labor analgesia in parturients. The optimal insertion of the epidural catheter is determined by several patient-related factors, including body mass index (BMI), gestational age, race, ethnic background, and the anatomy of the spine. To date, several studies have been conducted to evaluate the lumbar epidural space depth in varied populations across the globe. However, limited data are available regarding the BMI and its correlation with the skin-to-lumbar epidural distance (SLED) in Indian parturients. Hence, this observational study was undertaken to investigate the correlation between the BMI and the SLED under ultrasound guidance in parturients with 37 to 42 weeks of gestational age visiting the labor room of a tertiary care institute in central India.

Methods

This observational study was conducted from January 2023 to December 2023 at the All India Institute of Medical Sciences (AIIMS), Raipur. Informed and written consent was acquired from all the participants before enrolling them in the study. Parturients with 37 to 42 weeks of gestational age, aged 20-40 years, belonging to the American Society of Anesthesiologists physical status 2, were included. Patients who refused to give consent, with a diagnosed mental disorder, intellectual disabilities, pre-eclamptic/eclamptic patients, or those with spine deformity were excluded. Baseline patient parameters, including age, height, weight, and gestational age, were recorded, and BMI was calculated. Patients were categorized into different BMI classes, as classified by the World Health Organization. Ultrasound imaging of the L3-L4 interspace was done. An in-built caliper of the ultrasound machine was used to measure the SLED. The mean SLED in each BMI category was noted. The primary outcome of the study was to determine the correlation between BMI and SLED. A P-value of less than 0.05 was regarded as statistically significant.

Results

Two hundred patients were included in the final analysis. The BMI (kilogram/meter²) of the patients ranged from 19.67 to 40.00, with the mean ± standard deviation (SD) of 28.40 ± 4.35 and the median interquartile range (IQR) of 27.60 (25.20-31.24). The SLED of the participants in centimeters (cm) ranged from 3.12 to 5.17, with the mean ± SD of 4.01 ± 0.42 and median (IQR) of 4.03 (3.82-4.18). The SLED in five groups of BMI was not normally distributed; therefore, the Kruskal-Wallis test was used to make group comparisons, and a significant difference was noted (χ2 = 159.650, P < 0.001). The data regarding BMI and SLED among the patients were normally distributed. Pearson's correlation was used to explore the correlation between the two variables, and a strong positive correlation was noted with a correlation coefficient (r) = 0.94 and P < 0.001.

Conclusion

The ultrasound may be incorporated as a useful tool in routine clinical practice in the care of parturients to determine the SLED before the actual insertion of the epidural needle. This approach may reduce the number of attempts at lumbar epidural catheter placement. It may be particularly helpful in obese parturients, in whom it is difficult to identify the anatomical landmarks of the spine by palpation.

## Introduction

Placement of lumbar epidural catheters is the gold standard practice for providing labor analgesia in parturients [1]. The optimal insertion of the epidural catheter is determined by several factors, one of which is the variability in the depth of the epidural space, which may be influenced by the patient's weight, height, body mass index (BMI), gestational age, race, ethnic background, and spine anatomy [2]. To date, several studies have been conducted to evaluate the depth of the lumbar epidural space in varied populations across the globe [3-6]. These studies have shown an increase in the lumbar epidural space depth over the past few decades, from the mean depths of 4.2-4.9 cm in the 1980s and 1990s [5-7] to about 5.4 cm in 2011 [2], which mirrors the rising trend of obesity globally, and parturients are not an exception to it. Literature shows that both ethnicity and BMI significantly influence the skin-to-lumbar epidural distance (SLED). Moreover, at any given BMI, the SLED differs among ethnic groups. As compared to the Asian and Chinese parturients, the SLED is markedly higher in Black, British Black, and White parturients [2].

However, limited data are available regarding the BMI and its correlation with the skin to lumbar epidural distance (SLED) in Indian parturients. Moreover, no such data is available from central India. Hence, this observational study was undertaken to evaluate the correlation between the BMI and the SLED under ultrasound guidance in parturients with 37 to 42 weeks of gestational age (term parturients) visiting the labor room of a tertiary care institute in central India. The primary outcome was to assess the correlation between BMI and SLED in these patients.

## Materials and methods

Settings and design

The principles of the Declaration of Helsinki (2013) [8] and the guidelines of the Institute Ethics Committee (IEC) were followed in the conduct of this study. After obtaining approval from the IEC (2478/IEC-AIIMSRPR/2022, dated September 26, 2022) and registering the trial prospectively in the Clinical Trials Registry-India (CTRI/2023/01/048672, dated January 2, 2023), this observational study was conducted from January 2023 to December 2023 at AIIMS, Raipur, which is a tertiary care institute in central India.

Participants

Parturients with 37 to 42 weeks of gestational age, aged 20 to 40 years, belonging to the American Society of Anesthesiologists (ASA) physical status classification 2, were included [9,10]. Patients who refused to give consent, patients with a diagnosed mental disorder, patients with intellectual disabilities, pre-eclamptic patients, eclamptic patients, and patients with spine deformities were excluded from the study.

**Figure 1:**
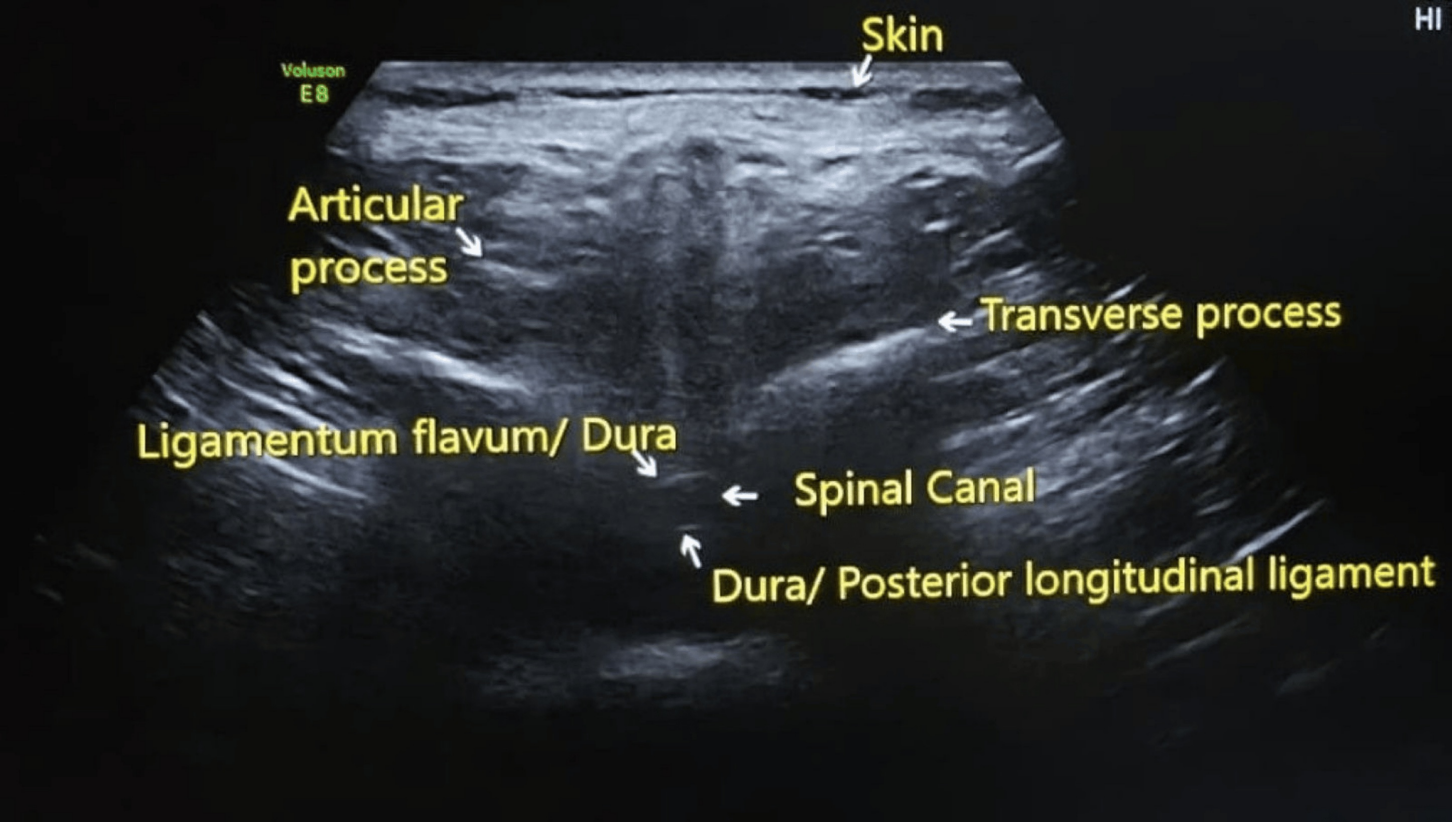
Ultrasound image of L3-L4 spine

Parturients visiting the labor room were assessed for eligibility using a purposive sampling technique. Informed and written consent was acquired from all the patients before enrolling them in the study. Baseline patient parameters, including age in years, height in meters (m), weight in kilograms (kg), and gestational age in weeks, were recorded, and BMI (kg/m²) was calculated [11]. To avoid any subjective bias, the SLED in all the patients was measured by a single observer who was trained in the use of ultrasound. The patients were made to sit comfortably. The L3-L4 interspace was identified by palpating the highest point of the iliac crest [12,13]. Ultrasound imaging of the L3-L4 interspace was done by a Mindray UMT-150 mobile trolley ultrasound machine (Mindray Bio-Medical Electronics Co., Ltd., Shenzhen, China). The curvilinear probe operating at a frequency of 2-5 megahertz was placed transversely, perpendicular to the long axis of the spine [14,15], and then adjusted slowly to visualize the L3-L4 intervertebral space. After identifying the L3-L4 intervertebral space, the ligamentum flavum-duramater unit was determined (Figure 1). The SLED was measured in centimeters (cm) using an in-built caliper of the ultrasound machine, from the skin to the internal surface of the ligamentum flavum-duramater unit [16,17].

Patients were categorized into different BMI classes, as classified by the World Health Organization (Table 1) [11]. The mean SLED in each BMI category was noted, and the findings were correlated.

**Table 1:** Classification of patients according to their BMI as per the recommendations of the WHO BMI, body mass index; kg, kilograms; m, meters; WHO, World Health Organization [11]

Classification	BMI (kg/m²)
Underweight	<18.50
Normal weight	18.50-24.99
Overweight	≥25.00
Pre-obese	25.00-29.99
Obese class I	30.00-34.99
Obese class II	35.00-39.99
Obese class III	≥40.00

Statistical analysis

Microsoft Excel spreadsheet, version 365 (Microsoft Corp., Redmond, WA) was used to record the data. The IBM SPSS Statistics for Windows, version 23 (IBM Corp., Armonk, NY) was used for data analysis. Mean ± standard deviation (SD) and/or median with interquartile range (IQR) were used to express continuous variables, whereas number (n) and percentage (%) were used to express categorical variables. Data were presented graphically wherever applicable. Skewness, kurtosis, and the Shapiro-Wilk test were applied to verify the normality of the data. A parametric test (Pearson’s correlation coefficient) was used to compute the correlation for data that were normally distributed. The non-parametric test (Kruskal-Wallis test) was used to make group comparisons for the data that were not normally distributed. A P-value of less than 0.05 was regarded as statistically significant.

Sample size 

Sample size calculation was based on the SD for the mean ultrasound depth and the minimum difference between the means in the different BMI patients, taken from a previous study done by Arzola et al. [18], using the following formula: \[n = \frac{2 \times (Z_{\alpha} + Z_{\beta})^2 \times \sigma^2}{\delta^2}\] where n is the sample size; Zα=2.58 at a 99% confidence interval; Zβ=0.84 at 80% power; σ=0.68 (SD); and δ=0.25 (minimum difference in the mean ultrasound depth). After substituting the values, a sample size of 173 was obtained, which was rounded off to 200.

## Results

**Figure 2:**
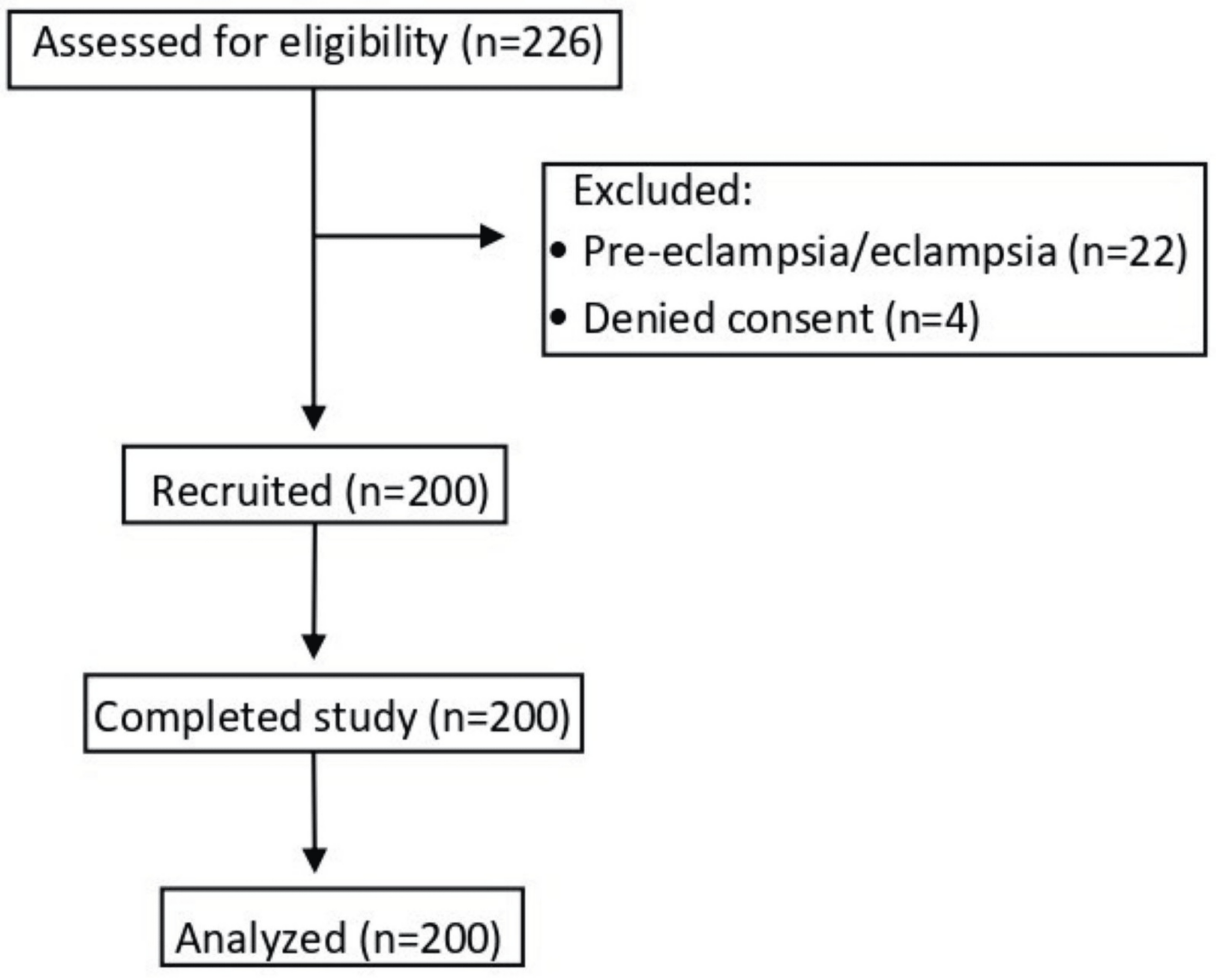
Flow diagram

Two hundred twenty-six patients were assessed for eligibility. Following exclusion, 200 patients were included in the final analysis (Figure 2).

Eighty-two (41%) patients belonged to the age group of 26-30 years, comprising the largest number. Sixty-five (32.5%) and 49 (24.5%) patients belonged to the age groups of 31-35 and 20-25 years, respectively. Only four (2%) patients belonged to the 36 and 40 years of age group, comprising the smallest number. None of the patients belonged to the underweight category with a BMI of <18.50 kg/m². Forty-six (23%) patients belonged to the normal weight category with a BMI of 18.50-24.99 kg/m². Eighty-seven (43.5%) patients belonged to the pre-obese group, with a BMI of 25.00-29.99 kg/m², whereas 50 (25%), 15 (7.5%), and 2 (1%) patients belonged to the obese class I, II, and III, respectively (Table 2).

**Table 2:** Distribution of patients in terms of age group and BMI The data has been represented as number and percentage BMI, body mass index; kg, kilograms; m, meters; n, number; %, percentage

Parameters	Number (n=200)	Percentage (%)
Age group (years)		
20-25	49	24.5%
26-30	82	41.0%
31-35	65	32.5%
36-40	4	2.0%
BMI (kg/m²)		
<18.50	0	0
18.50-24.99	46	23.0
25.00-29.99	87	43.5
30.00-34.99	50	25.0
35.00-39.99	15	7.5
≥40.00	2	1.0

The age of the patients (in years) ranged from 20 to 37, with a mean ± SD of 28.65 ± 3.80 and a median (IQR) of 29.00 (26.00-32.00). The gestational age of the patients (in weeks) ranged from 37.00 to 40.86, with a mean ± SD of 38.42 ± 0.94 and a median (IQR) of 38.29 (37.57-39.04). The weight of the patients (in kg) ranged from 47 to 102, with a mean ± SD of 69.58 ± 11.84 and a median (IQR) of 69.00 (60.00-76.00). Patient’s height (in meters) ranged from 1.40 to 1.75, with a mean ± SD of 1.56 ± 0.06 and a median (IQR) of 1.56 (1.52-1.61). The BMI of the patients (in kg/m²) ranged from 19.67 to 40.00, with a mean ± SD of 28.40 ± 4.35 and a median (IQR) of 27.60 (25.20-31.24). The SLED of the patients (in cm) ranged from 3.12 to 5.17, with a mean ± SD of 4.01 ± 0.42 and a median (IQR) of 4.03 (3.82-4.18) (Table 3).

**Table 3:** Demographic and clinical characteristics The data have been represented as mean ± SD, median (IQR) and range (min.-max.) BMI, body mass index; SLED, skin to lumbar epidural distance; SD, standard deviation; IQR, interquartile range; min., minimum; max., maximum; kg, kilograms; m, meters; cm, centimeters

Parameters	Mean ± SD	Median (IQR)	Range (min.-max.)
Age (years)	28.65 ± 3.80	29.00 (26.00-32.00)	20.00-37.00
Gestational age (weeks)	38.42 ± 0.94	38.29 (37.57-39.04)	37.00-40.86
Weight (kg)	69.58 ± 11.84	69.00 (60.00-76.00)	47.00-102.00
Height (m)	1.56 ± 0.06	1.56 (1.52-1.61)	1.40-1.75
BMI (kg per m²)	28.40 ± 4.35	27.60 (25.20-31.24)	19.67-40.00
SLED (cm)	4.01 ± 0.42	4.03 (3.82-4.18)	3.12-5.17

On comparing the SLED among various BMI groups (Table 4), it was noted that with the increase in the BMI, the mean and median SLED were also increasing, with the lowest reading in the BMI group of 18.50-24.99 kg/m² and the highest reading in the BMI group of ≥ 40.0 kg/m². The SLED in five groups of BMI was not normally distributed; therefore, a non-parametric test (Kruskal-Wallis test) was used to make group comparisons, and a significant difference was noted (χ2 = 159.650, P < 0.001).

**Table 4:** Association between BMI and SLED The data have been represented as mean ± SD and median (IQR) BMI, body mass index; SLED, skin to lumbar epidural distance; kg, kilograms; m, meters; cm, centimeters; SD, standard deviation; IQR, interquartile range A P-value of less than 0.05 was considered statistically significant *Kruskal-Wallis Test

BMI (kg/m²)	SLED (cm)		P value^*^
	Mean ± SD	Median (IQR)	
18.50-24.99	3.47 ± 0.24	3.42 (3.25-3.66)	<0.001
25.00-29.99	3.99 ± 0.13	3.97 (3.92-4.07)	
30.00-34.99	4.28 ± 0.20	4.22 (4.13-4.37)	
35.00-39.99	4.80 ± 0.18	4.81 (4.69-4.94)	
≥ 40.00	4.98 ± 0.23	4.98 (4.9-5.07)	

With regard to the distribution of BMI and SLED among the patients, there appeared to be only one mode/peak, making the data unimodal. Two out of the three normality assumption criteria, namely, skewness, kurtosis, and the Shapiro-Wilk test results, were suggestive of normality, so it appeared that the data were normally distributed. Pearson's correlation (a parametric test) was used to explore the correlation between BMI and SLED, and a strong positive correlation was noted with a correlation coefficient (r) = 0.94 and P < 0.001.

**Figure 3:**
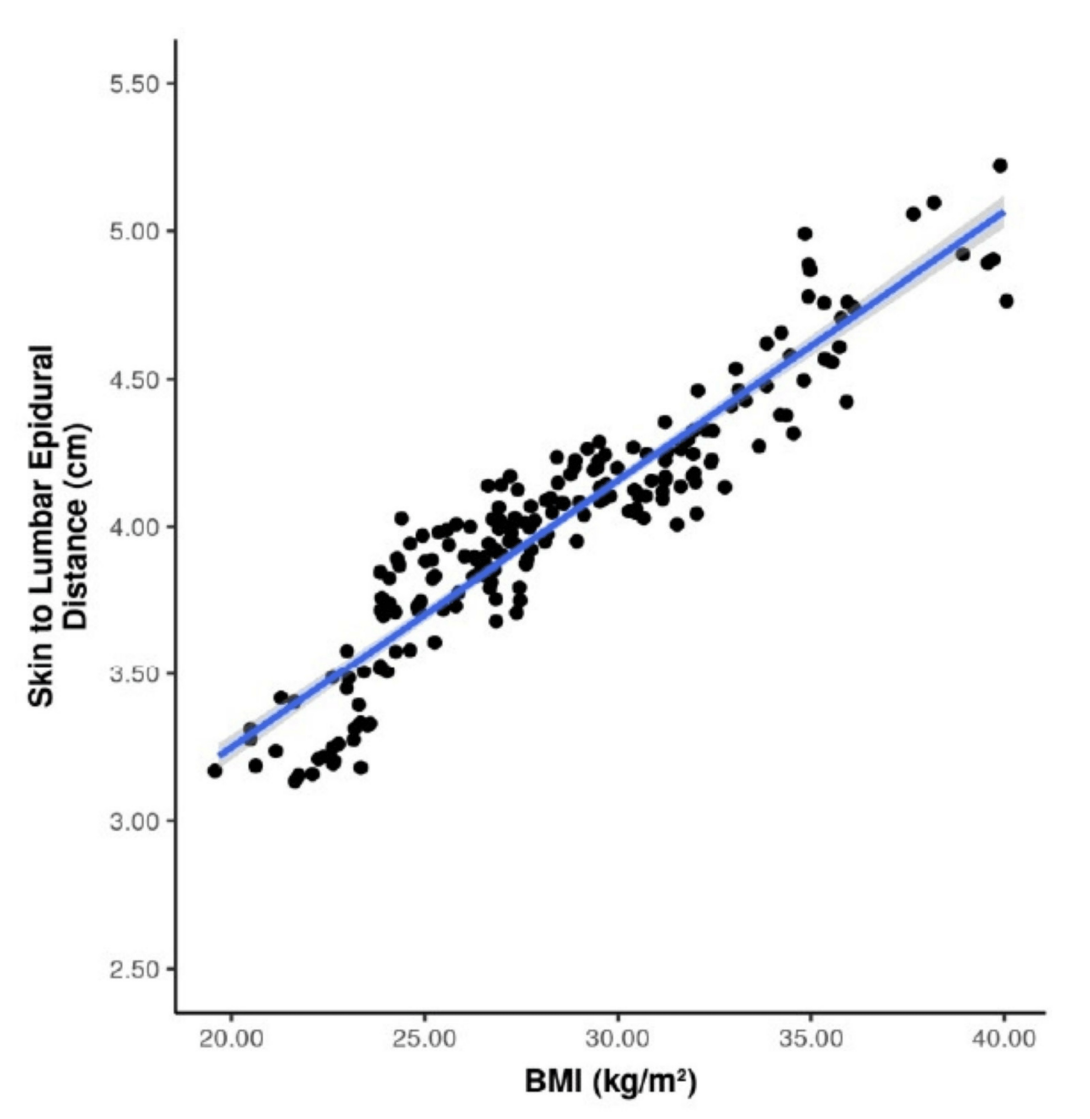
Scatterplot depicting the correlation between BMI and SLED BMI, body mass index; SLED, skin to lumbar epidural distance; kg, kilograms; m, meters; cm, centimeters

Figure 3 illustrates a scatter plot demonstrating the relationship between BMI (kg/m²) and SLED (cm). Each dot corresponds to an individual participant. The blue regression line indicates the overall trend between the two parameters, while the grey band represents the 95% confidence interval of the fitted line.

## Discussion

Two hundred patients were included in the final analysis. Patients were categorized into different BMI classes as classified by the World Health Organization [11]. As there were no participants in the BMI category of <18.50 kg/m², the data from the remaining five BMI groups were analyzed and assessed for their correlation with SLED (cm).

In our study, a strong positive correlation was observed between BMI and SLED. The findings of our study were similar to those of Clinkscales et al., Sharma et al., and Al-Saeed et al. [1,2,19]. Clinkscales et al. conducted a study at the Women's Hospital in Michigan to study the correlation between SLED and BMI [1]. They analyzed records of 2009 parturients with gestational age of 36 weeks and beyond and noted the mean lumbar epidural space depth to be 5.3 ± 1.21 cm and the mean BMI (kg/m²) to be 31.5 ± 6.2. The authors concluded that increasing BMI is associated with increasing depth of the lumbar epidural space. Sharma et al. collected data from 1210 parturients from various maternity units in the United Kingdom to establish the influence of ethnicity and BMI on the SLED and noted the mean SLED to be 5.4 ± 1.1 cm [2]. The authors noted a significant influence of BMI and ethnicity on SLED and concluded that the SLED increased with a corresponding increase in BMI. Al-Saeed et al. conducted a study on 255 parturients at Al-Mosawy Hospital, Basrah, comprising parturients from Iraqi, Japanese, Chinese, Pakistani, German, and British ethnicities, to find the correlation between SLED and BMI [19]. The authors noted that the SLED in most Iraqi obstetric parturients in Basrah ranged from 3 to 7 cm, with a mean of 4.43 ± 0.96 cm, which was greater than that reported in Japanese, Chinese, and Pakistani parturients but lower than that reported in British and German women. The authors concluded that the SLED has a significant correlation with the BMI. In our study, the mean SLED was 4.01 ± 0.42 cm, which was in close approximation to the findings of Al-Saeed et al. [19]. Considering the similar ethnicity of the Indian and the Pakistani women, the mean SLED of our study was almost similar to that of the Pakistani parturients from the aforementioned study. Arzola et al. conducted a study on 61 Canadian parturients to assess the reliability of the ultrasound as a screening tool to ease insertion of lumbar epidurals. In their study, the mean BMI (kg/m²) was 29.7 ± 4.8 [18]. The mean SLED (cm) in terms of ultrasound depth and needle depth was 4.66 ± 0.68 and 4.65 ± 0.72, respectively. The authors found a good agreement between the ultrasound-determined depth and the actual needle depth and suggested that the ultrasound can be used as a dependable screening tool to ease the insertion of lumbar epidurals in laboring patients. In our study, the mean BMI (kg/m²) was 28.40 ± 4.35, while the mean SLED (cm) was 4.01 ± 0.42, which is in close proximity to the findings of Arzola et al. [18]. The main difference between our study and that done by Arzola et al. was that in our study, we assessed SLED using ultrasound only, whereas Arzola et al. assessed the SLED in terms of ultrasound depth as well as the needle depth [18]. Hamza et al. did an observational study in 2123 obstetric patients in a French hospital receiving lumbar epidurals for labor analgesia and cesarean section to identify the factors influencing SLED [20]. The authors noted a positive correlation of the SLED with the parturient's weight and BMI. The SLED was higher when an epidural catheter was inserted in the lateral position as compared to that in the sitting position. The authors noted that the SLED is influenced by the weight and position of the patient.

Strengths

Data regarding SLED in parturients from the Indian sub-continent are limited. So, our study was robust in the sense that it provided us with data from the Indian parturients. Secondly, to avoid any subjective bias, the SLED in all the patients in our study was measured by a single observer trained in the use of ultrasound, which also adds to the strengths of the study.

Limitations

There were a few limitations to this study. Firstly, it was conducted at a single tertiary care center located in central India; so, results may differ in the case of a multicenter study catering to populations from other geographic regions of India. Secondly, it was conducted on term parturients, aged 20-40 years, so the data obtained from this study cannot be applied to other patient populations, including pediatric, male, non-pregnant female, and pre-term obstetric patients. Thirdly, the measurement of SLED using ultrasound was done in a sitting position; so, the results obtained from this study may not be applicable for the insertion of lumbar epidurals in the lateral position. Further, only ultrasound-guided SLED was measured in our study, which was not confirmed with the actual needle insertion. Lastly, cost-effectiveness may limit the use of ultrasound in resource-limited countries like India.

## Conclusions

In our study, a strong positive correlation was noted between the BMI (kg/m²) and SLED (cm) in parturients when measured under ultrasound guidance in the sitting position.

The ultrasound may be incorporated as a useful tool in routine clinical practice in the care of parturients to determine SLED before the actual insertion of the epidural needle. This approach may reduce the number of attempts at lumbar epidural catheter placement. It may be particularly helpful in obese parturients, in whom it is difficult to identify the anatomical landmarks of the spine by palpation.
